# A sight on the current nanoparticle-based gene delivery vectors

**DOI:** 10.1186/1556-276X-9-252

**Published:** 2014-05-21

**Authors:** Solmaz Maleki Dizaj, Samira Jafari, Ahmad Yari Khosroushahi

**Affiliations:** 1 Biotechnology Research Center, Tabriz University of Medical Sciences, Tabriz, Iran; 2 Student Research Committee, Tabriz University of Medical Sciences, Tabriz, Iran; 3 Department of Pharmaceutical Nanotechnology, Faculty of Pharmacy, Tabriz University of Medical Sciences, Tabriz, Iran; 4 Drug Applied Research Center, Tabriz University of Medical Sciences, Tabriz, Iran; 5 Department of Pharmacognosy, Faculty of Pharmacy, Tabriz University of Medical Sciences, Daneshgah Street, P.O.Box 51664, 14766 Tabriz, Iran

**Keywords:** Gene delivery, Non-viral vectors, Nanoparticles, Inorganic vectors

## Abstract

Nowadays, gene delivery for therapeutic objects is considered one of the most promising strategies to cure both the genetic and acquired diseases of human. The design of efficient gene delivery vectors possessing the high transfection efficiencies and low cytotoxicity is considered the major challenge for delivering a target gene to specific tissues or cells. On this base, the investigations on non-viral gene vectors with the ability to overcome physiological barriers are increasing. Among the non-viral vectors, nanoparticles showed remarkable properties regarding gene delivery such as the ability to target the specific tissue or cells, protect target gene against nuclease degradation, improve DNA stability, and increase the transformation efficiency or safety. This review attempts to represent a current nanoparticle based on its lipid, polymer, hybrid, and inorganic properties. Among them, hybrids, as efficient vectors, are utilized in gene delivery in terms of materials (synthetic or natural), design, and *in vitro*/*in vivo* transformation efficiency.

## Review

### Introduction

Gene therapy is described as the direct transfer of genetic material to cells or tissues for the treatment of inherited disorders and acquired diseases. The base of this therapeutic method is to introduce a gene encoding a functional protein altering the expression of an endogenous gene or possessing the capacity to cure or prevent the progression of a disease [1-3]. This method was initially performed in 1990 in the USA for the treatment of adenosine deaminase-deficient SCID (combined immune deficiency disease) patients [4]. This therapy is not only used in genetic deficiencies, but also in other complicated diseases, such as viral infection (human immunodeficiency virus), autoimmunity (rheumatoid arthritis), cancer, diabetes, coronary, and artery disease [5]. With the progress of this technique, gene therapy will become an effective therapeutic method for neurodegenerative conditions, hemophilia, AIDS, asthma, and the myriad of other genetic and acquired diseases that affect humanity [2].

By considering the mentioned issues, the choice of a suitable method for DNA delivery to the targeted cells beseems very important at the point of receiving appropriate genes. Although gene therapy can be carried out using naked DNA into the target cells, having negative nature of cellular membrane and negative charge of large DNA molecules, the nucleic acid-based therapeutics cannot cross cellular membranes by simple passive diffusion methods. Hence, to facilitate the transfer of DNA molecules into a cell, the existence of a vector is necessary [6,7]. Viral and non-viral vectors, two major types of vectors for gene delivery, are currently being utilized in clinical trials at similar levels.

In gene delivery, it is relatively common to follow biomimetic approaches. Biological systems include modified viruses and mildness bacteria. Viral vectors are more efficient than non-viral vectors for DNA delivery but may present a significant risk to patients, while non-viral carriers are inherently safer than viral carriers [8-10]. Furthermore, in contrast to the viral gene delivery systems, the non-viral carriers are expected to be less immunogenic, with simple preparation and a possible versatile surface modification [7]. The non-viral vectors are usually made of lipids or polymers with/without using other inorganic materials where they can also be prepared from a lipid-polymer or lipid-polymer-inorganic hybrid. The choice of gene delivery strategies among several delivery systems depend on some factors including the improvement of vectors, kind of expression systems, and better understanding of molecular biology of target site and employing of the advances in the identification of new genes and new targets [11]. Recently, nanotechnology approaches play an important role in the design novel and efficient non-viral gene delivery vectors. In this review, we will focus on introducing lately synthesized nanoparticles as vectors with gene delivery applications.

### Non-viral vectors

In considering the viral gene delivery vector safety concerns regarding the risk of excessive immune response (adenovirus) and insertion mutagenesis (retroviruses), the use of non-viral vectors can overcome the mentioned safety problems [12]. Non-viral vectors either consist of natural vectors (plasmid DNA or small nucleic acids, antisense oligonucleotides, small interfering RNAs) or synthetic vectors (liposomes, cationic polymers) [11]. Naked DNA, usually in plasmid form, is the simplest form of non-viral transferring of a gene into a target cell [13-16]. Because of low transferring efficiency of a bare plasmid, several physical (electroporation, ultrasound, gas-filled micro-bubbles) and chemical (liposomes) approaches have been exploited to enhance their transformation efficiency [17].

In another type of classification, non-viral delivery vectors can be categorized as organic (lipid complexes, conjugated polymers, cationic polymers, etc.) and inorganic (magnetic nanoparticles, quantum dots, carbon nanotubes, gold nanoparticles, etc.) systems [18].

Among the materials used to design non-viral vectors, attention has recently increased on the natural biomaterials due to their unique properties such as biodegradability, biocompatibility, and controlled release.

**Figure 1:**
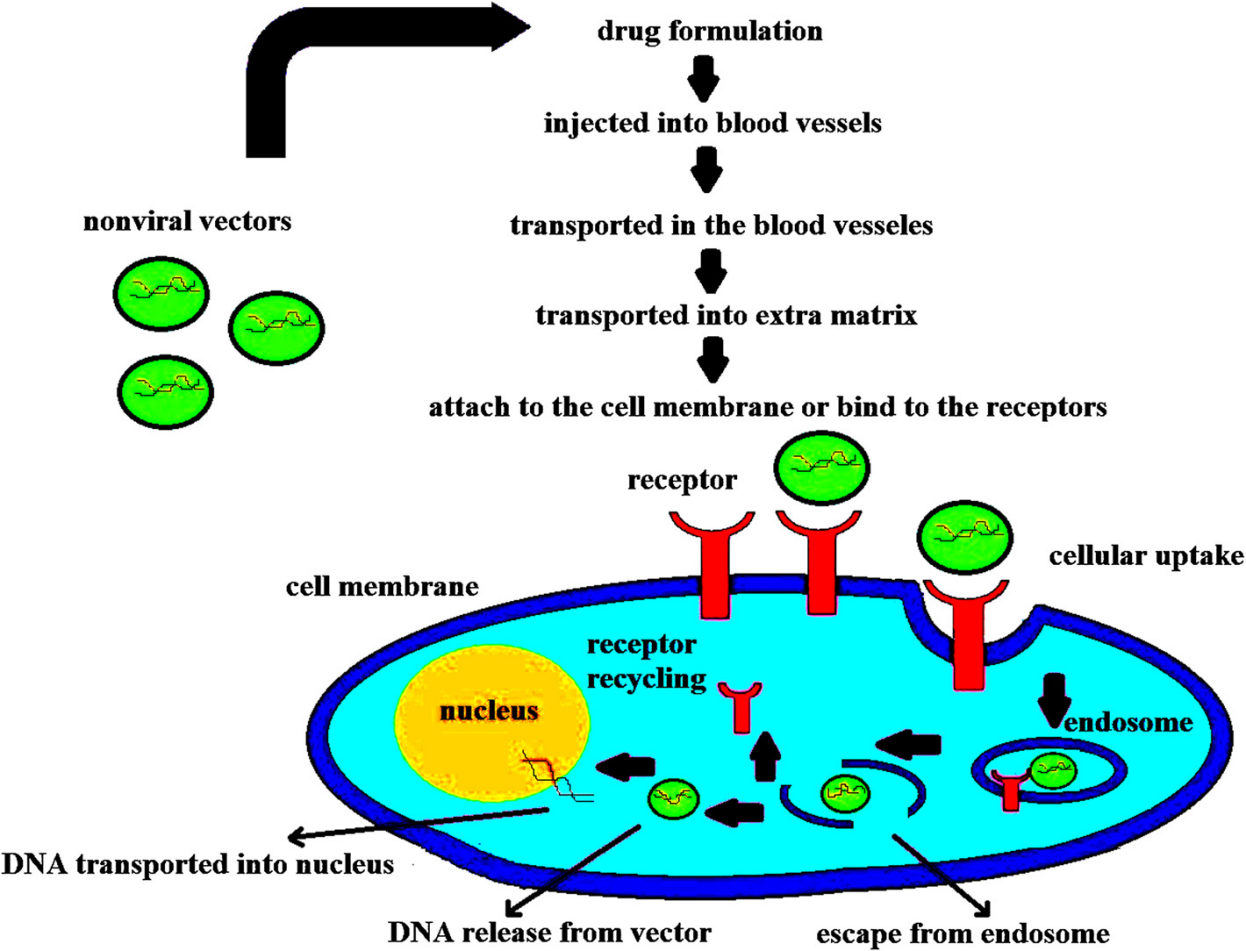
Internalization of non-viral vectors into cell and passage to nucleus through cytoplasm following endocytosis.

The delivery carriers necessitate being small enough to be internalized into the cells and enter the nucleus passing through the cytoplasm and escaping the endosome/lysosome process following endocytosis (Figure 1). The use of nanoparticles in gene delivery can provide both the targeted and sustained gene delivery by protecting the gene against nuclease degradation and improving its stability [19-22].

### Nanoparticles in gene delivery

In the field of nanomedicine, nanotechnology methods focus on formulating therapeutic biocompatible agents such as nanoparticles, nanocapsules, micellar systems, and conjugates [22,23]. Nanoparticles are solid and spherical structures ranging to around 100 nm in size and prepared from natural or synthetic polymers [24]. To reach the large-size nucleic acid molecule, the cytoplasm, or even the nucleus, a suitable carrier system is required to deliver genes to cells which enhance cell internalization and protect the DNA molecule from nuclease enzymatic degradation (e.g., virosomes, cationic liposomes, and nanoparticles). To achieve the suitable carrier system, the nanoparticles can be considered as a good candidate for therapeutic applications because of several following reasons: (1) They exist in the same size domain as proteins,(2) they have large surface areas and ability to bind to a large number of surface functional groups, and (3) they possess controllable absorption and release properties and particle size and surface characteristics [25]. Nanoparticles can also be coated with molecules to produce a hydrophilic layer at the surface (PEGylation) to increases their blood circulation half-life. Poloxamer, poloxamines, and chitosan have also been studied for surface modifications. The surface modifications are performed for different objectives: (1) the utilized groups can usually block the electrostatic and hydrophobic interactions thus protect nanoparticles from opsonization and (2) targeting of tumors or organs accordingly increases selective cellular binding and internalization through receptor-mediated endocytosis [24].

Nanoparticles exploited in gene delivery were categorized into four major groups in this investigation for further explanations.

#### Lipid-based nanoparticles

Cationic liposomes, cationic lipids, cationic solid lipids, and cationic emulsions are lipid-based structures routinely utilized for nucleic acid delivery to cells. Cationic lipids are positive amphiphilic molecules with four main constituents: (1) the cationic polar head group, which has the important role in the self-assembly interaction with DNA, (2) a hydrophobic chain that affects the physical properties of the lipid bilayer (such as flexibility and therefore gene transfer efficiency), (3) a spacer between two mentioned sections that influences the determination of chemical stability, biodegradability and gene transfection efficiency, and (4) a backbone (often glycerol-based) domain as a scaffold. A large number of cationic lipids have previously been utilized in gene delivery, such as quaternary ammonium detergents, cationic derivatives of cholesterol and diacylglycerol, lipid derivatives of polyamines. Dioleylpropyl trimethylammonium chloride (DOTMA) and dioleoyl trimethylammonium propane (DOTAP) are two of the most popular cationic lipids [20].

A cationic liposome is a liposome composed of a positive and a helper lipid which can protect DNA from enzymatic degradation in blood circulation and can interact with the negatively charged cell membrane to probably facilitate cell internalization. Compared to viral vectors, liposomes possess some preferred properties such as safe preparation, toxicity monitoring, and risk reduction of immunological problems by controlling their size using ultrasonication or extrusion through porous membranes with specific pore sizes.

Cationic solid lipid core-shell structures were composed from high melting point lipids as core and surfactants as covered shell. These structures have low transformation efficiency and slight risk of toxicity at high-dose applications which are considered as promising vectors for systemic administrations. The solid lipid nanoparticles (SLNs) can condense DNA into nanometric colloidal particles and able to transfect mammalian cells under *in vitro* conditions. Comparisons between cationic lipids and cationic polymers illustrated some advantages for SLNs such as (1) a relative ease of production without requirements for organic solvents, (2) the possibility of large scale production with qualified production lines, and (3) good storage stabilities together with the possibility of steam sterilization and lyophilization [20,26,27].

Cationic emulsions were constructed using a hydrophobic oil phase covered by the cationic lipid. These cationic emulsions possess remarkable advantages such as their nanosized range, biocompatibility, biodegradability, physical stability, and low toxicity which make them as favorable carriers for delivering gene to the targeted cells. Furthermore, easy and low cost productions of cationic emulsions provide the scaling-up possibility for emulsion-based systems [20,28].

#### Polymer-based nanoparticles

Cationic polymers are one of the most significant non-viral gene delivery systems. These polymers have positively charged groups in their backbone and can interact with the negative charge of anionic genetic materials [29]. Cationic polymers can bind to DNA molecules to form neutralized, nanometer-sized complexes known as polyplexes. Polyplexes have some advantages compared to lipoplexes (complex of lipids-DNA) such as small size, narrow distribution, higher protection against enzymatic degradation, more stability, and easy control of the physical factors. Although, the *in vivo* efficacy of polymeric gene delivery is low, using of biomaterials for gene delivery can reduce many of the safety concerns with viral gene delivery [25,29].

Due to their unique properties such as biodegradability, biocompatibility, and controlled release, natural biopolymers and proteins have recently increased attention in gene delivery. Biopolymers are polymers produced by living organismsand can be categorized in three groups: polysaccharides, proteins, and nucleic acids. To fabricate nanoparticles from these biopolymers, for therapeutic objects, a variety of materials have been used [25]. Naturally derived proteins such as collagen, elastin, and fibronectin have been used in biomaterial nanoparticle fabrication. Silk proteins due to their properties such as slow biodegradability, biocompatibility, self-assembling property, excellent mechanical property, and controllable structure and morphology are promising materials as biomaterial nanoparticles [25]. Collagen, the main component of extracellular matrix, is one of the main biomaterials in fabrication of gene delivery nanoparticles due to biocompatibility, low antigenicity, and biodegradability. Collagen can be formed to hydrogels without the use of chemical crosslinking, but additional chemical treatment is necessary for prepared nanoparticles due to their weak mechanical strengths [23,25]. Collagen is often chosen as a biomaterial because this protein is abundant in the animal kingdom and plays a vital role in biological functions, such as tissue formation, cell attachment, and proliferation [30]. In addition, proteins such as albumin, β-casein, and zein are good candidates for fabrication of nanoparticles due to their non-immunogenicity, non-toxicity, biodegradability, and biocompatibility [29]. Albumin can be considered an ideal material as a delivery carrier due to its remarkable properties including high binding capacity, high stability in pH and heat, preferential uptake in tumor and inflamed tissue, biodegradability, low toxicity, low immunogenicity, and suitable blood circulation with a half-time of 19 days [29,31]. Beta casein, the major milk protein, can self-assemble into micellar structure by intermolecular hydrophobic interactions. Therefore, its micellar structure is a suitable feature for the delivery applications. Zein is an alcohol-soluble protein existence in corn with properties such as biocompatibility, low water uptake value, high thermal resistance, and good mechanical properties. The main application of zein is in edible coating for foods and pharmaceuticals. Zein exists as small nanosized globules and consists of both hydrophobic and hydrophilic amino acid residues; therefore, it has been applied as a promising carrier system [23,25,29].

Polysaccharides, long carbohydrate molecules of repeated monosaccharide units, are another group of biopolymers. Examples of them consist of chitosan, alginate, heparin, hyaluronic acid, pullulan, and dextran. The cationic polyelectrolyte nature of chitosan provides a strong electrostatic interaction with mucus, negatively charged mucosal surfaces, and other macromolecules such as DNA [32]. Besides, the presence of primary amine groups in the structure of chitosan caused this biodegradable, biocompatible, and non-toxic biopolymer to be used as an appealing vector for non-viral genes [33]. It is capable of forming stable, small (20 to 500 nm) particles with complex pDNA and its binding efficiency relate to the molecular weight and the degree of deacetylation [25]. It has better protection against DNase degradation and higher biocompatibility compare to polymers such as polyethyleneimine (PEI).

The literatures have shown the physicochemical characteristics of chitosan complexes, such as size, charge, and complexation efficiency with nucleic acid, are affecting factors in overcoming physiological and cellular barriers to gene delivery [34]. The transfection efficiency of chitosan started slower but increased over time with lowering cytotoxity results for *in vivo* cases.

Polysaccharides and their derivatives are used for biomedical applications due to high stability, biocompatibility, and main of all biodegradability. Three types of celebrated polysaccharide nanoparticles have been identified by cross-linking, polyion complex, and self-assembly [25].

Sometimes, the hybrid of protein and polysaccharide can be used to fabricate nanoparticles for gene delivery. Albumin-chitosan-DNA-based core-shell nanoparticles are investigated for gene delivery objectives. The studies of these nanoparticles showed that they have higher biocompatibility and less toxicity compared to poly-l-lysine (PLL) and PEI. Additionally, their core-shell structure provides two separate parts for gene delivery [31].

Not only natural protein- or polysaccharide-based nanoparticles, but also synthetic polymer nanoparticles have been also paid high attention. Protein-mimicked polypeptide-based nanoparticles are unique features of proteins, and today, a number of them have been synthesized. They have properties such as well-defined composition, monodisperse molecular weight and potential biocompatibility. These nanoparticles often were fabricated with a sequence of Val-Pro-Gly-Xaa-Gly by self-assembly of elastin-like polypeptides (ELP) [25]. Peptide conjugated to antibody has been used for delivery of siRNA to T cells of humanized mice to suppress HIV infection [35].

PEI polymers are able to successfully complex DNA molecules and they also have distinct transfection efficiency in a wide variety of cell types compared to some other polymer systems described later. PEI derivatives cross-linked with different acrylates showed high gene expression in the lung or the spleen in mice. They also showed only little toxicity in cell culture experiments [36]. *In vivo* application of this polymer promises to take the polymer-based vector to the next level where it can undergo clinical trials and then could be used for delivery of therapeutics in humans [37]. PLL is another cationic polymer, and its efficiency in gene delivery depends on its molecular weight. In low molecular weight, its complex with DNA is less soluble and rapidly removed by the Kupffer cells of the liver. With increasing the molecular weight, the efficiency of PLL is enhanced, interestingly [38].

Dendrimers are three-dimensional polymers with spherical, highly branched structures. Frequently used dendrimers are polyamines, polyamides, or polyesters. Because of its high transfection efficiency, polyamidoamine (PAMAM) is the most commonly used. The type of amine groups and the size of dendrimers have an influence on their transfection efficiency. The primary amine groups promote DNA cellular uptake because of their participation in DNA binding but the buried tertiary amino groups act as a proto-sponge in endosomes and enhance the release of DNA into the cytoplasm. The studies show that with increasing the size and diameter, dendrimers enhance transfection efficiency [39,40]. Recently, nitrogen-core poly(propyl ether imine) (PETIM) dendrimer DNA complexes have been investigated and results showed low toxicities and efficient gene delivery vector properties. Quantitative estimation, using luciferase assay, showed that the gene transfection was at least 100 times higher when compared to poly(ethyleneimine) branched polymer, having similar number of cationic sites as the dendrimer [40].

Poly lactic-co-glycolic acid (PLGA)-based nanoparticles have been recognized as a potential vector to deliver genes. They are used in gene therapy for tumor and other miRNA-related diseases such as diabetes and cardiovascular and neurodegenerative diseases. The researches show that PLGA makes an improved safety profile in comparison with high-molecular weight PEIs and liposome. Also, it is demonstrated that serum cannot inhibit the transfection activity of these nanoparticles [41]. PLGA nanoparticles are internalized in cells through pinocytosis (fluid phase) and also through clathrin-based endocytosis. These nanoparticles rapidly escape the endo-lysosomes and enter the cytoplasm within 10 min of incubation [24]. Nanoparticles consisting of a PLGA core and a chitosan coating (with a positive charge) can form stable complexes with DNA or RNA. Researchers showed that in contrast to pure PLGA particles, the active groups localized on the surface of the carrier caused the fast release [7].

Polyion complex micelles (PICs) are core-shell structures of polyplex. Initially, Kataoka et al. introduced PIC micelles using PLL-PEG block copolymer by which PLL segments and pDNA formed a hydrophobic core by electrostatic interactions and PEG played a role as a surrounding hydrophilic shell layer [42]. Due to the use of PEG, PICs have both the higher transfection and the longer circulation half-life compare to polyplexes. PIC micelles have some noticeable properties compared to conventional polyplex and lipoplex systems such as excellent colloidal stability in protein aqueous media, high solubility in aqueous media, high tolerance toward nuclease degradation, minimal interaction with biological components, and prolonged blood circulation. Also, in these systems, with functionalization of PEG group in the shell, the probability of targeting modification is enhanced [43]. Thiol-decorated polyion complex micelles prepared through complexation between PEG-*b*-poly(2-(*N*,*N*-dimethylamino)ethyl methacrylate) and a 20-mer oligonucleotide have been investigated in this area [44,45].

One main concern about polymeric nanoparticles in gene delivery is coupling of the interior and exterior composition of them with polymer backbone and affects all the functions and biophysical properties of the polymer/DNA particles. One proposed method is coating poly(glutamic acid)-based peptide to the exterior composition of a core gene delivery particle to change their function under *in vivo* conditions [46].

#### Inorganic nanoparticles

Several inorganic nanoparticles mainly including carbon nanotubes (CNTs), magnetic nanoparticles, calcium phosphate nanoparticles, gold nanoparticles, and quantum dots (QDs) are routinely utilized as gene delivery carriers. These nanoparticles possess many advantages in gene delivery. According to reports, they are not subjected to microbial attack and show also good storage stability [47].

The use of carbon nanotubes (CNTs) in *in vitro* applications has been of interest but their potential for *in vivo* use is limited by their toxicity. Due to their nanometer needle structure, CNTs can easily cross the plasma membrane using an endocytosis mechanism without inducing cell death [18]. Single-walled nanotubes have been exploited to deliver CXCR_4_ and CD_4_-specific siRNA to human T cells in HIV infections [35]. Use of CNTs for biomedical applications is limited due to their low biocompatibility. Surface modification or functionalization can increase solubility in aqueous solutions and biocompatibility [48]. According to reports, functionalized single-walled nanotubes (SWNTs) can facilely enter human promyelocytic leukemia (HL60) and T cells [49]. This ability can be used to deliver bioactive protein or DNA into mammalian cells.

Fullerenes, especially C60, are water-insoluble allotropes of carbon. Low solubility of fullerenes in the cell medium can be improved by addition of dimethylformamide (DMF) but it will lead to increased cytoxicity and consequently reduced cell viability [50]. Recently, Isobe et. al. (2010) reported that due to their positive charge and their cationic nature, aminofullerenes have the capability of transfection with formation fullerene/DNA complexes [51].

Magnetic nanoparticles have been proven to be effective in gene delivery particularly in cardiovascular diseases. These particles are submicron-sized synthetic particles that respond to magnetic field. In the magnetic drug/gene delivery system, the gene directly binds to the magnetic particle or carrier. Magnetic nanoparticles can be dispersed in a polymer matrix (generally silica, polyvinyl alcohol (PVA) or dextran) or encapsulated within a polymer or metallic shell. Targeted gene delivery can be done by attaching different types of functionalized groups such as carboxyl groups, amines, biotin, streptavidin, antibodies, and polyethyleneimine (particularly *in vitro* uses) to shell or matrix. The recent research showed that transfection time is significantly reduced for magnetic nanoparticles in comparison to that for non-viral agents and that magnetic nanoparticles have been used to successfully deliver small interfering RNA and antisense oligonucleotides under *in vitro* and *in vivo* conditions [29,52]. Recently, magnetic calcium phosphate nano-formulations have been used for transfection of DNA [53]. The DNA-loaded magnetic system in A549 and HepG2 tumor cells indicated that the magnetic nano-formulation could improve the targeted gene delivery for cancer therapy with under an external magnetic field.

Metallic nanoparticles, especially GNPs, have the advantages that they are easy to prepare, have high gene transfection efficiency, and their surfaces are very amenable to chemical modification [54]. Because of low chemical reactivity and unique stability of gold, this metal is very attractive as coating for magnetic nanoparticles. Also, functionalization of the gold surface with thiol groups, allows the linkage of functional ligands and subsequently make the materials suitable for catalytic and optical applications [55,56].

Calcium phosphate nanoparticles, routinely used for *in vitro* transfection, have been investigated as a powerful non-viral gene delivery [57]. These nanoparticles alone, or in combination with other vectors (viral or nonviral), show good gene delivery properties especially when incorporated in the colloidal particulate systems [58]. Indeed, divalent metal cations, such as Ca^+2^, Mg^+2^, Mn^+2^, and Ba^+2^ can form ionic complexes with the DNA thus give stabilized structures. The complexes can then be carried across cell membrane *via* ion channel-mediated endocytosis. Because of low transfection efficiency and inability to apply *in vivo* conditions, calcium phosphate can be considered as a more efficient material compared to other viral and non-viral vectors. These drawbacks can be overcome by preparing ultra-low size calcium phosphate nanoparticles entrapping DNA molecules [59,60]. Furthermore, calcium phosphate nanoparticles are very safe and can overcome many targeting problems such as an efficient endosomal escaping, rendering sufficient protection of DNA in the cytosol and providing an easy passage of cytosolic DNA to the nucleus [59]. These nanoparticles can be useful in gene delivery in the treatment of bone defects due to high calcium phosphate content of the bone [61]. It seems that the use of nanotubes, nanoshells, and mesoporous nanoparticles (such as silica mesoporous nanoparticle) is a promising idea for gene delivery because of their hollow and porous structures and facile surface fictionalization as well [62].

Recently, the application of silica nanoparticles has been reported as a non-viral vector for efficient *in vivo* gene delivery. Silica nanoparticles functionalized with amino groups can efficiently bind to plasmid DNA and protect it from enzymatic digestion and effect cell transfection *in vitro*. It has been shown that by loading of DNA on the modified silica nanoparticles, DNA has been protected from degradation by DNase which can effectively be taken up by COS-1 cells [63]. This type of silica nanoparticles overcomes many of the limitations of unmodified silica nanoparticles. Indeed the presence of organic group on the surface of these nanoparticles imparts some degree of flexibility to the otherwise rigid silica matrix and increases the stability of them in aqueous systems. Based on the previous investigation results, these nanoparticles as a non-viral gene delivery carriers have a promising future direction for effective therapeutic manipulation of the neural stem/progenitor cells as well as *in vivo* targeted brain therapy [12]. Functionalized dendrimer-like hybrid silica nanoparticles are attractive nanocarriers for the advanced delivery of various sized drugs and genes simultaneously because these nanoparticles have hierarchical pores, unique structure, large surface area, and excellent biocompability [64].

Quantum dot (QD) has been successfully applied for *in vitro* and *in vivo* transfection. QDs are nearly spherical semiconductor particles with core-shell structure. The semiconducting nature and the size-dependent fluorescence of these nanocrystals have made them very attractive for diagnosis of diseases. Gene-associated drugs can be loaded within a QD core or attached to the surface of these nanoparticles through direct conjugation or electrostatic complexation by which QDs can protect the gene from degradation by nucleases [65-67]. Super paramagnetic iron oxide nanoparticles (SPIONS) are utilized as gene delivery systems. In pulmonary gene delivery systems, either branched biodegradable polyesters or PEG-coated super paramagnetic iron oxide nanoparticles are promising carriers. These nanoparticles can maintain their transfection ability at *in vivo* condition after nebulization [68,69]. However, there are challenges, such as the standardization of therapy response and the stability of complex nanoparticles under certain biological conditions. SPION are known to be an excellent carrier for siRNA delivery because they are biocompatible and target-functionalized. In spite of hard-to-transfect cell lines, the novel method such as magnetofection can be used for delivering of SPION with plasmid DNA or siRNA, where these nanoparticles is subjected to oscillating magnetic fields that facilitate caveolae-mediated endocytosis of SPION and cargo nucleic acid [70]. Due to nano-dimension size and also stability, inorganic nanoparticles are being extensively used as promising gene carriers. All of reviewed studies signify that inorganic nanoparticles such as gold and silica possess attractive properties such as high fictionalization ability, good biocompatibility, low toxicity, and potential capability of targeted delivery [71]. Also, it seems that functionalized CNTs according to their large inner volume (that allows the loading of small biomolecules), quantum dots because of their unique luminescent properties, Calcium phosphate nanoparticles due to wide availability and high safety, and lately, SPIONs owing to their valuable magnetic properties are appropriate candidates as carriers for gene transfection.

#### Hybrid nanoparticles

**Figure 2:**
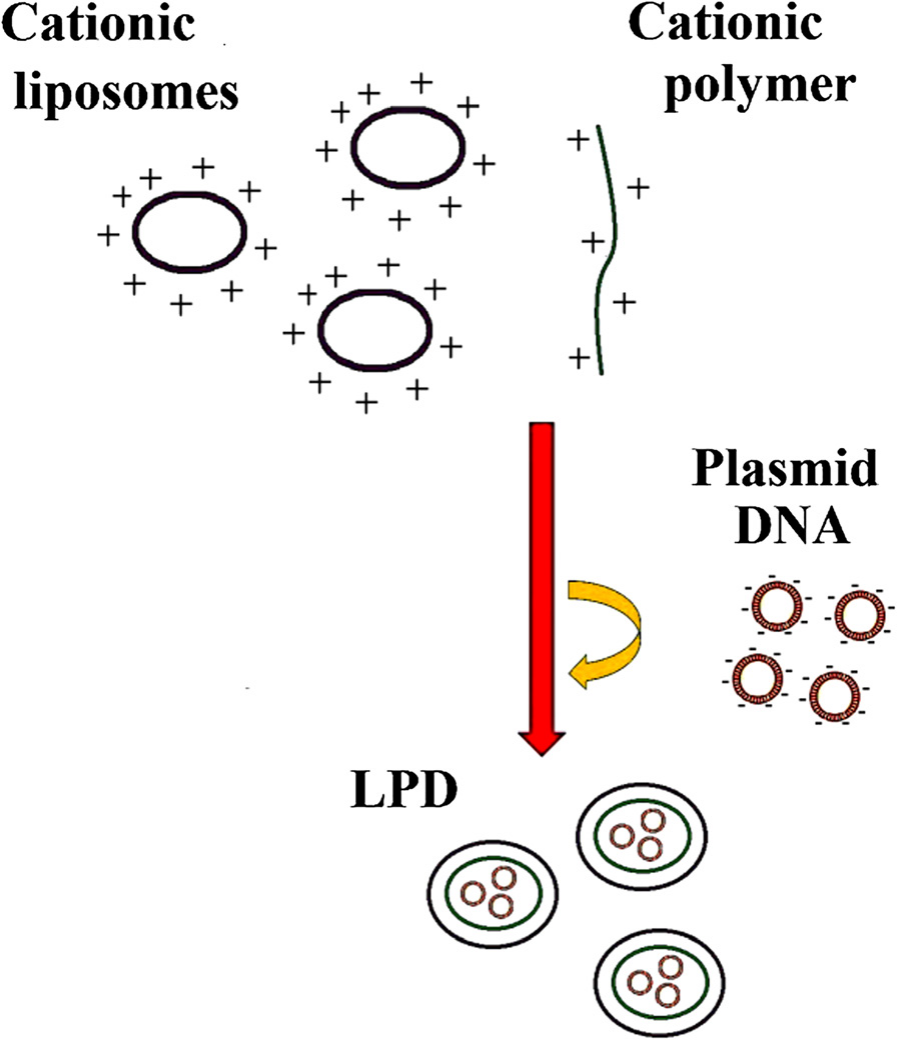
Schematic processes of LPD formation.

Hybrid nanoparticles can be categorized into two groups: liposome-polycation-DNA (LPD) nanoparticles and multilayered nanoparticles. LPD nanoparticles can be fabricated by spontaneous rearrangement of a lipid shell around a polycation-DNA core (Figure 2) [72].

Indeed, they are complexes which consist of liposomes (that are either made of cationic (LPDI) or anionic (LPDII) lipids) and polyplexes sometimes referred to as lipopolyplexes. Polycations, unlike cationic polypeptides such as poly-l-lysine, histone, and protamine can be condensing DNA in highly compressed structures in nanometric diameter. Formation of multilayered nanoparticles are carried out through layer-by-layer (LbL) assembly of polycations and polyanions (e.g., DNA). The properties of the self-assembled multilayers depend on the choice of their building blocks. Using of multifunctional gene vectors improve the loading dose of DNA cellular uptake, controlling the release of DNA and target delivery [25,73]. Some important properties and advantages/disadvantages of non-viral vectors are presented in Tables 1 and 2, respectively.

**Table 1:** Characterizations of nanoparticulate non-viral vectors in gene delivery

**Non-viral vectors**	**Transferring efficiency**	**Preparation**	**Cellular toxicity**	**Applications**	**References**
Inorganic nanoparticles	Low	Easy	Frequently toxic	*In vitro*	[18,48,56]
Polymer-based nanoparticles	Low	Easy	Low toxicity	*In vitro*	[25,29,31]
Lipid-based nanoparticles	Low	Difficult	Toxic (at high dose)	*In vitro*	[20]
Hybrid nanoparticles	High	Easy	Low toxicity	*In vitro*/*In vivo*	[25,73]

**Table 2:** Advantages and disadvantages of non-viral vectors

**Type of nanoparticle**	**Advantages**	**Disadvantages**	**References**
Inorganic nanoparticles	Short time of transfection, easy preparation, wide availability, rich functionality, high transfection efficiency, potential capability for targeted delivery and controlled release	Most of them are instable, toxic and non-biocompatible	[18,48,56]
Polymer-based nanoparticles	Small size, narrow distribution, more stability, high protection against enzymatic degradation, low toxicity and high cationic potential	Low biodegradability, low efficacy	[25,29,31]
Lipid-based nanoparticles	Safe preparation, low immunogenicity,	Toxicity at high dose, difficult preparation, low transformation efficiency	[20]
Hybrid nanoparticles	Improved the loading dose of DNA cellular uptake, controlling the release of the DNA and target delivery compared to other non-viral vectors	Toxicity at very high dose	[25,73]

## Conclusion

Gene delivery is one of the recent attractive therapeutic methods that involved viral and non-viral vectors. From the stability and safety point of view, non-viral vectors have more efficiency in gene transfection. Non-viral vectors can be efficiently passing through biological barriers compared to viral vectors. Among these vectors, nanoparticle carriers have been successfully applied for *in vitro* gene delivery and efforts continue in improving *in vivo* applications. While a wide variety of organic, inorganic, or hybrid materials are used for the production of nanoparticles, polymeric nanoparticles have great therapeutic application. Polymeric nanoparticles can be produced in a wide range of sizes and varieties and can be used in sustained and targeted gene delivery for long periods. Among the naturally derived and synthetic biomaterials for gene delivery, natural biopolymers can potentially avoid many of the safety concerns with viral gene delivery due to high biocompatibility, low toxicity, and good biodegradability. In conclusion, the utilization of biopolymers such as proteins, polypeptides, and polysaccharides will be one of the potentially promising methods for gene delivery. However, the clinical application of gene delivery still is in its initial stage, but more progression of it is expected in the near future.

## Competing interests

The authors declare that they have no conflicts of interests.

## Authors' contributions

SMD and SJ have made a significant contribution to the work or the drafting of the manuscript. AYK scientifically has revised and is the corresponding author of the manuscript. All authors read and approved the final manuscript.
